# Hair growth-promoting effect of *Geranium sibiricum* extract in human dermal papilla cells and C57BL/6 mice

**DOI:** 10.1186/s12906-017-1624-4

**Published:** 2017-02-13

**Authors:** William A. Boisvert, Miri Yu, Youngbin Choi, Gi Hee Jeong, Yi-Lin Zhang, Sunghun Cho, Changsun Choi, Sanghyun Lee, Bog-Hieu Lee

**Affiliations:** 1 John A. Burns School of Medicine, University of Hawaii, Honolulu, HI 96813 USA; 2 Department of Food and Nutrition, College of Biotechnology and Natural Resources, Chung-Ang University, Anseong, 17546 Korea; 3 Department of Integrative Plant Science, College of Biotechnology and Natural Resources, Chung-Ang University, Anseong, 17546 Korea

**Keywords:** Hair loss, Antioxidant, *Geranium sibiricum* L, Human dermal papilla cells, Transforming growth factor beta 1

## Abstract

**Background:**

*Geranium sibiricum* L. has been used as a medicinal plant to treat diarrhea, bacterial infection, and cancer in Bulgaria, Peru, and Korea. However, its hair growth-promoting effect was not investigated so far. This study examined the effects of *Geranium sibiricum* L. extract (GSE) on hair growth, using in vitro and in vivo models.

**Methods:**

Antioxidant, proliferation and migration assay of GSE was performed with human dermal papilla cells (hDPCs). Hair-growth promoting effect was measured in animal model. Relative expression of interleukin-1, vascular endothelial growth factor, hepatocyte growth factor, and transforming growth factor beta 1 was determined by real time RT-PCR. Expression of Ki-67 and stem cell factor were analyzed by immunohistochemistry.

**Results:**

GSE treatment proliferated and migrated human dermal papilla cells (hDPCs) more than treatment of 10 μM minoxidil. GSE significantly stimulated the expression of Ki-67 protein and the mRNA levels of hepatocyte growth factor and vascular endothelial growth factor in hDPCs. Topical application of 1,000 ppm GSE for 3 weeks promoted more significant hair growth on shaved C57BL/6 mice than did 5% minoxidil. The histological morphology of hair follicles demonstrated an active anagen phase with the induction of stem cell factor. GSE treatment significantly reduced the number of mast cells and the expression of transforming growth factor beta 1 in mouse skin tissues.

**Conclusions:**

These results demonstrated that GSE promotes hair growth in vitro and in vivo by regulating growth factors and the cellular response.

## Background

Hair loss is defined as a state in which hair does not exist at a typical area or less hair regrowth is observed in the area [1]. In modern society, hair loss occurs via genetic reasons as well as external factors such as environmental pollution, work stress, and alteration of hormone secretion [1]. Minoxidil and finasteride are the only chemicals approved by the US Food and Drug Administration to treat hair loss [2–4]. However, both these chemicals have serious adverse effects such as weight gain, edema, angina pectoris, and hypogonadism in men and can lead to the birth of deformed baby if used by pregnant women. In efforts to find natural substances that are less toxic than minoxidil and finasteride, previous studies have screened about 1,000 plant extracts for hair growth or hair loss-preventing effects [5, 6]. Among the natural extracts, *Allium cepa* extract and *Ziziphus jujuba* extract were found to promote hair growth [5, 6], with the antioxidant capacity of each extract being concluded as the contributing factor.

All living organisms are constantly challenged by a diversity of exogenous- and endogenous stressors, which induce biological responses to protect or adapt to stressors. The systemic biological response of the organism to stressor induces stress response through activation of hypothalamic-pituitary-adrenal axis (HPA) by proinflammatory cytokines to increase circulating glucocorticoids and catecholamines [7]. The growing body of evidence now supports that a wide range of neuropeptides, neurotransmitters, and neurohormones modulating systemic stress responses can indeed alter hair growth, indicating that hair follicles represent an important target for stressors [8].

Plant phenolics and flavonoid are recently of interest, since these compounds possess antioxidation, anti-inflammatory, anti-microbial, and anti-carcinogenic properties [9]. *Geranium sibiricum* L., which belongs to the Geraniaceae family of plants, grows in China, Japan, Korea, and some European countries. While it is used as a food ingredient in Russia and Turkey, it has been used as a medicinal plant to treat diarrhea, bacterial infection, and cancer in Bulgaria, Peru, and Korea [10]. The extract and phenolic compounds from *G. sibiricum* showed high antioxidant capacity in 1,1-diphenyl-2-picrylhydrazyl (DPPH) radical scavenging, superoxide radical scavenging, nitric oxide scavenging, β-carotene-linoleic acid bleaching, and reducing power [11]. As several pharmacological studies of *G. sibiricum* have shown anti-inflammatory, anti-bacterial, anti-diarrheal effect and anti-gastric ulcer action [12–15], it is widely used in cosmetic industry nowadays. Shim et al. [16] has reported that ethanol extract of *Geranium sibiricum* L. decreased expression of interleukin (IL)-1β, COX-2 and inducible nitric oxide synthase (iNOS) in PMACI stimulated HMC-1 cells. IL-1β and COX-2 are known as potent inhibitors of hair growth in vitro and in vivo. Inui et al. [17] has also found that dihydrotestosterone (DHT), contributing to androgenic alopecia, increases iNOS from occipital dermal papilla cells and suggested that iNOS and NO are downstream effectors of androgen receptors. However, the effects of *G. sibiricum* extract (GSE) on hair growth have not been studied so far. Therefore, the study aimed to investigate whether the topical treatment of GSE could promote hair growth in vitro and in vivo models by regulating the expression of growth factors and inflammatory cytokines.

## Methods

### Preparation of GSE and HPLC analysis

GSE was purchased from a Korea Plant Extract Bank at Korea Research Institute of Bioscience & Biotechnology (KRIBB, Daejeon, Korea). *G. sibiricum* L. used in this study were collected from Mountain Gamak in Paju-si, Korea. Botanical samples were authenticated by Professor Shin-Ho Kang from Department of Natural Medicine Resources in Semyung University. The air-dried plant materials were ground into fine powder and extracted with 100% methanol. After filtration of total extract, the extract was evaporated to dryness in vacuum and weighed. The recovery rate was 6.7%.

GSE was dissolved in dimethylsulfoxide (DMSO). Water, acetonitrile (ACN), and methanol (MeOH) used in this research were of HPLC grade, and all other reagents were of analytical grade. Corilagin and gallic acid were provided by Professor Sam Sik Kang, Seoul National University, Korea. They were used as standard chemical for high pressure liquid chromatography (HPLC) analysis. HPLC chromatograms were recorded with a Waters Breeze system (Massachusetts, USA) equipped with a Waters 1525 binary HPLC pump and 2489 system UV/VIS detector.

HPLC separation of corilagin and gallic acid for qualitative and quantitative analysis was performed using a reverse phase system. Discovery^®^ C18 (4.6 × 250 mm, 5 μm) column was used with a mobile phase consisting of a gradient system of water containing 0.2% acetic acid and ACN (90:10 to 60:40 for 30 min). UV detection was conducted at 270 nm. The injection volume was 10 μl and the flow rate was 1 mL/min. All injections were performed in triplicate. Corilagin and gallic acid were weighed and dissolved in MeOH to obtain a stock standard solution (1.0 mg/mL). Solutions of aqueous corilagin and gallic acid were prepared at concentrations of 0.0001, 0.001, 0.01, 0.1, and 1 mg/mL for the construction of a calibration curve. The contents of the analysis were determined from the corresponding calibration curves. The calibration functions of the compound were calculated using the peak area (Y), concentration (X, μg/10 μl), and mean values (*n* = 3) ± standard deviation (SD).

### Antioxidative activity of GSE

The analysis of total polyphenol content was determined using Folin-Denis method. Point five milliliter of GSE prepared 50% methanol at concentration of 0.2 mg/mL was mixed with 0.5 mL of Folin-Denis reagent (Fluka, Buchs, Switzerland) and was added to 0.5 mL of 10% sodium carbonate solution after 3 min at room temperature. The mixture was kept at room temperature for 1 h and then the absorbance was measured at 760 nm using 7315 UV spectrophotometer (JENWAY, Staffordshire, United Kingdom). Total polyphenol content was expressed as mg of tannic acid (Yakuri Pure Chemicals Co., Ltd., Kyoto, Japan) equivalent (TAE).

To determine the total flavonoid content, 0.5 mL of the 0.2 mg/mL dissolved in methanolic GSE was mixed with 5 mL of diethylene glycol (Duksan Pure Chemicals, Ansan, Korea). After 5 min at room temperature, the mixture was added to 0.5 mL of 1 N NaOH (Duksan Pure Chemicals, Ansan, Korea) and allowed to stand for 1 h in 37 °C water bath. The absorbance was measured at 420 nm using 7315 UV spectrophotometer (JENWAY, Staffordshire, United Kingdom). Total flavonoid content was expressed as mg of naringin (Tokyo Kasei Kogyo Co., Ltd., Tokyo, Japan) equivalent (NE).

The method was conducted as described by Blois [18] with some modifications to determine DPPH scavenging activity of GSE. 0.5 mL of diluted to 0.2 mg/ mL GSE was mixed with 3 mL of 0.2 mM methanolic DPPH solution. 0.5 ml of 100% methanol was used as a carrier control. After 30 min in the dark at room temperature, the absorbance of the mixture was measured at 517 nm by a 7315 UV spectrophotometer (JENWAY, Staffordshire, United Kingdom). The electron donating ability (EDA) was calculated by the following equation.

$$ E D A\ \left(\%\right) = \frac{AB{S}_{control} - A B{S}_{sample}}{AB{S}_{control}} \times 100 $$

### Proliferation and migration assay of human dermal papilla cells (hDPCs)

Cell proliferation was determined by Cell Counting Kit-8 (CCK-8) (Sigma, St. Louis, MO, USA). hDPCs were seeded at a density of 5 × 10^3^ cells/well of a 96-well microplate. After incubation for 24 h, the cells were cultured with 100 μL of serum-free Dulbecco’s modified Eagle’s medium (DMEM, Sigma) for 24 h and then treated with 9.8, 19.5, 39.1, 78.1, 156.3 μg/mL of GSE in DMEM. Serum-free DMEM medium and triton X-100 (Sigma, St. Louis, MO, USA) were used as negative control (NC) and blank, respectively. Boyera et al. [19] has found that micromolar concentrations of minoxidil stimulated proliferation in both human keratinocytes of epidermal and hair follicle origin cell types and in all culture conditions, whereas millimolar concentrations inhibited cell growth. Similarly, treatment concentration of minoxidil was examined for hair growth effect with less toxicity in our preliminary study. Thus, 10 μM minoxidil was used as positive control (PC) in vitro assay. After treatment for 24 h, 10 μL of CCK-8 solution was added to each well, the cells were then incubated at 37 °C for 3 h. The absorbance was measured at 450 nm (test wavelength) and 650 nm (reference wavelength) by microplate spectrophotometer (Epoch Multi-Volume Spectrophotometer System, BioTek, VT, USA). The measured absorbance was used to determine cell proliferation by the following equation.

$$ \mathrm{Cell}\ \mathrm{proliferation}\ \left(\%\right)=\frac{{\left({\mathrm{ABS}}_{\mathrm{sample}}\hbox{-} {\mathrm{ABS}}_{\mathrm{blank}}\right)}_{450\mathrm{nm}}\hbox{-} {\left({\mathrm{ABS}}_{\mathrm{sample}}\hbox{-} {\mathrm{ABS}}_{\mathrm{blank}}\right)}_{650\mathrm{nm}}}{{\left({\mathrm{ABS}}_{\mathrm{control}}\hbox{-} {\mathrm{ABS}}_{\mathrm{blank}}\right)}_{450\mathrm{nm}}\hbox{-} {\left({\mathrm{ABS}}_{\mathrm{control}}\hbox{-} {\mathrm{ABS}}_{\mathrm{blank}}\right)}_{650\mathrm{nm}}}\times 100 $$

Migration assay was conducted as describe by previous study [20]. hDPCs were plated on 8-well chamber slide (Nalge Nunc International, Naperville, Illinois, USA) and grown to 90% confluence in 10% FBS DMEM medium. The cells were then scratched in straight line with 200 μL pipette tip and the cell debris was removed by washing the cells with sterile PBS. hDPCs were treated with 9.8, 19.5, 39.1, 78.3 μg/mL of GSE. Serum-free DMEM medium and 10 μM minoxidil were used as NC and PC, respectively. After incubation for 24 h, hDPCs were fixed with 4% paraformaldehyde, washed two times with phosphate buffered saline (PBS). The cells were stained with hematoxylin (Sigma, St. Louis, MO, USA) and images were taken using a Leica 500 optical microscope (Leica, Wetzlar, Germany) at a magnification of 40 ×.

### Animal care and in vivo experiment for hair growth

Fifteen male 4-week-old C57BL/6 mice were purchased from Central Lab, Animal Inc. (Seoul, Korea). The mice were acclimated to their surroundings for 2 weeks to lead to the early onset of the telogen phase in mice hair cycles. Mice were randomly assigned to the experimental groups with 5 mice per group and were singly housed in cages to minimize the loss of the topically applied extracts by contact with other mice. All animals were bred in a laminar airflow room with 12 h of artificial light and darkness for 1 week. Animal facility was maintained at 22 ± 1 °C room temperature and a relative humidity of 55 ± 10%. All experimental procedures were approved by the Animal Care Committee of the Chung-Ang University (approval no: 13–0058).

Experimental groups contained NC, PC, and GSE group. Mice were randomly separated into three groups with five mice per group. In order to synchronize the stage of hair growth, back skin of all experimental animals were artificially shaved. Topical treatments on the shaved back skin were applied daily with 1% dimethyl sulfoxide (NC group), 5% minoxidil (dissolved into 1% DMSO) (PC group), or GSE dissolved in 1% DMSO with the final concentration of 1,000 ppm. Hair growth of each mouse was measured and photographed every week for 3 weeks after shave.

### Histopathology of hair growth

Dorsal skin tissues from each mouse were excised and fixed in 4% neutral formalin for 24 h. They were embedded in paraffin after routine tissue processing. For the histopathogic evaluation of hair follicles, 4 μm tissue section was stained with standard hematoxylin and eosin (H&E) protocol. For the counting of mast cells, toluidine blue stain (Sigma, St. Louis, MO, USA) was performed on skin tissues by manufacturer’s protocol. The morphology of hair follicles, the number of mast cells, and expression of stem cell factor (SCF) were evaluated by veterinary pathologists.

### Immunohistochemistry of Ki-67 and stem cell factor

To confirm the effect of GSE on hDPCs proliferation, the immunohistochemistry for Ki-67 was performed with some modification described in previous study [21]. hDPCs were seeded at a density of 2 × 10^3^ cells/well of an 8-well chamber slide and incubated until grow to 80-90% confluence in 10% FBS DMEM medium. After culture with serum-free DMEM medium for 24 h, the cells were added to each treatment and incubated for 24 h; serum-free DMEM (NC), 10 μM minoxidil (PC), 19.5 μg/mL of GSE. The cells were fixed for 20 min with 4% paraformaldehyde, washed three times with PBS. Endogenous alkaline phosphatase was quenched with levamisole and 10% normal goat serum was used for blocking non-specific reaction. Monoclonal mouse anti-human Ki-67 antibody (DAKO, Glostrup, Denmark) at 1:100 was incubated for 1 h at room temperature. Polyclonal goat anti-mouse immunoglobulins/alkaline phosphatase (DAKO, Glosturp, Denmark) was applied for 1 h. Positive signals were visualized with freshly prepared fast red (Vector Laboratories Inc., Burlingame, California, USA). Images were taken using a Leica DM 500 optical microscope (Leica, Wetzlar, Germany) at a magnification of 200 ×.

In order to examine the expression of the stem cell factor in skin tissues, immunohistochemistry was performed by standard ABC protocol. Mouse anti-stem cell factor antibody (Santa cruz, Dallas, TX, USA) at 1:100 dilutions was incubated. Secondary antibody used with polyclonal goat anti-mouse immunoglobulins/horse radish peroxidase (DAKO, Glosturp, Denmark). For the substrate of horse radish peroxidase, 3,3’-diaminobenzidine (DAB; Roche Diagnostics GmbH, Mannheim, Germany) was used to visualize the positive signal on tissues. Each well was mounted with coverslip using aqueous mounting media (Vector laboratories).

### mRNA relative expression of cytokines

In vitro model, hDPCs were plated on 6-well plate (3 × 10^4^) and incubated until grow to 80-90% confluence in 10% FBS DMEM medium. After culture with serum-free DMEM medium for 24 h, The cells were added to each treatment and incubated for 24 h; serum-free DMEM (NC), 10 μM minoxidil (PC), 19.5 μg/mL of GSE. Total RNA was extracted using RNeasy Mini kit (Qiagen, Dusseldorf, Germany) according to manufacturer’s instruction. In vivo model, mouse skins of each group were collected after 3 weeks of depilation. Total RNA of skin tissue was extracted using RNeasy Mini kit (Qiagen) following the manufacturer’s instruction.

For analyzing the expression of hepatocyte growth factor (HGF), vascular endothelial growth factor (VEGF), and transforming growth factor-beta 1 (TGF-β1) of hDPC or mouse skin, 1 μg of total RNA was treated with DNase I (Invitrogen). cDNA of mRNA was synthesized using cDNA synthesis kit (Thermo Fisher Scientific Inc., Waltham, MA, USA). Quantitative real-time polymerase chain reaction (qPCR) was conducted with Piko-real 96 real-time PCR system (Thermo Fisher Scientific Inc., Waltham, MA, USA) and performed for 45 cycles at 95 °C for 15 s, 60 °C for 30 s, 72 °C for 30 s. cDNA was amplified using Maxima SYBR Green/ROX qPCR Mater Mix 2X (Thermo Fisher Scientific Inc., Waltham, MA, USA). Primers were used as follows: glyceraldehyde 3-phosphate dehydrogenase (GAPDH), 5′-GGA AGG TGA AGG TCG GAG TC-3′ (forward); GAPDH, 5′-CTC AGC CTT GAC GGT GCC ATG-3′ (reverse); VEGF, 5′-CTT TAG AGA TCA GCC CAA CC-3′ (forward); VEGF, 5′-CTA CCC AGA GGG AAG AAA TAA C-3′ (reverse); HGF, 5′-AGA AAT GCA GCC AGC ATC AT-3′ (forward); HGF 5′-CAC ATG GTC CTG ATC CAA TC-3′ (reverse), TGF-β1; 5′-GCC CTG GAC ACC AAC TAT TG-3′ (forward); TGF-β1; 5′-GTC CAG GCT CCA AAT GTA GG-3′ (reverse).

### Statistical analysis

Data are presented as mean ± standard error (SE). One-way analysis of variance (ANOVA) and Duncan’s multiple range test was performed using SPSS program for windows, version 21.0 (SPSS Inc., Chicago, USA). Statistical significance was defined as *p* < 0.05.

## Results

### Active components of GSE

**Fig. 1:**
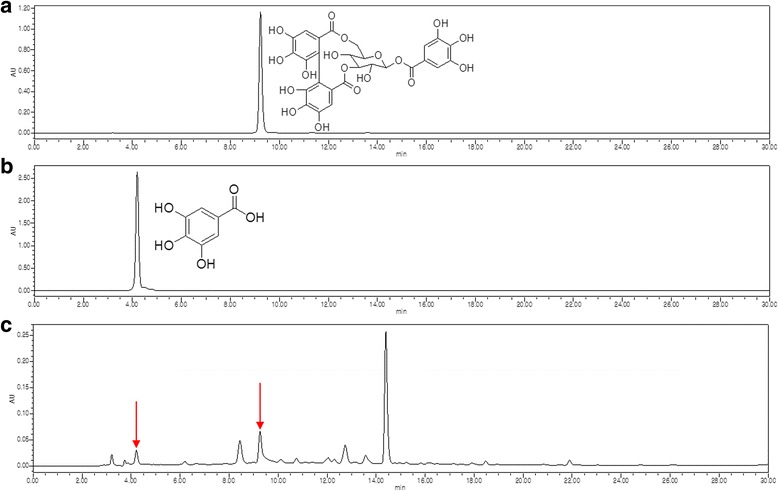
HPLC chromatograms of corilagin (**a**), gallic acid (**b**), and GSE (**c**)

Contents of GSE were analyzed by HPLC (Fig. 1). The corilagin and gallic acid contents of GSE were quantified using the linear regression equation. The linear regression data from the extracts had a good linear relationship and the resulting equation was valid over the relevant concentration range. The linear calibration equations were Y = 917496X + 24289 and Y = 2000000X + 91670, where Y is peak area and X is concentration of corilagin and gallic acid. The correlation coefficients (*r* ^2^) of corilagin and gallic acid were 0.9998 and 0.9997, respectively. The contents of corilagin and gallic acid were determined as 47.33 ± 1.99 and 3.31 ± 0.33 mg/g of GSE, respectively (Fig. 1).

### Antioxidative activity

In order to compare the antioxidant capacity of GSE, its total polyphenol and flavonoid contents and EDA were measured. Total polyphenol, flavonoid and EDA of GSE were 305.9 mg TAE/g extract, 104 mg NE/g extract and 76.6%, respectively.

### Proliferation and migration of hDPCs

**Fig. 2:**
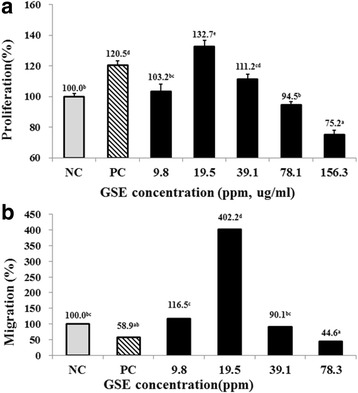
Effect of GSE on the proliferation and migration of human dermal papilla cells (hDPCs) (**a**) Proliferation of hDPCs was analyzed by the Cell Counting Kit-8 assay, and values are shown as the mean with SEM. **b** Migration effect of GSE was measured by the scratch migration assay, and the alteration of the scratch line width was converted into a percentage. NC: negative control of hDPCs treated with Dulbecco’s modified Eagle’s medium; PC: positive control of hDPCs treated with 10 μM minoxidil

Hair comprises at least fifteen distinct types of cells where the dermal papilla cell (DPC) is a primary cell type that regulates hair growth. Thus, determining the proliferation and migratory effects of GSE on hDPCs is necessary. The proliferative and migratory effects of GSE (9.8-156.3 ppm) were measured in hDPCs (Fig. 2a). The treatment of 19.5 ppm of GSE showed the highest proliferation rate of hDPCs. The proliferation rate of hDPCs was even higher in 19.5 ppm of GSE than PC by 132.7% vs. 120.5%. The concentration of 19.5-39.1 ppm GSE was significantly higher in proliferation rate than NC (*p* < 0.05). It was observed that GSE exhibited toxicity (cell viability less than 85%) above the concentration of 156.3 ppm.

On the other hand, the migration rate of the PC group was 59%, whereas that of 19.5 ppm GSE-treated group was 402%, indicating 19.5 ppm GSE-treated group had the highest migration rate among all the treatment groups (*p* < 0.05) (Fig. 2b).

**Fig. 3:**
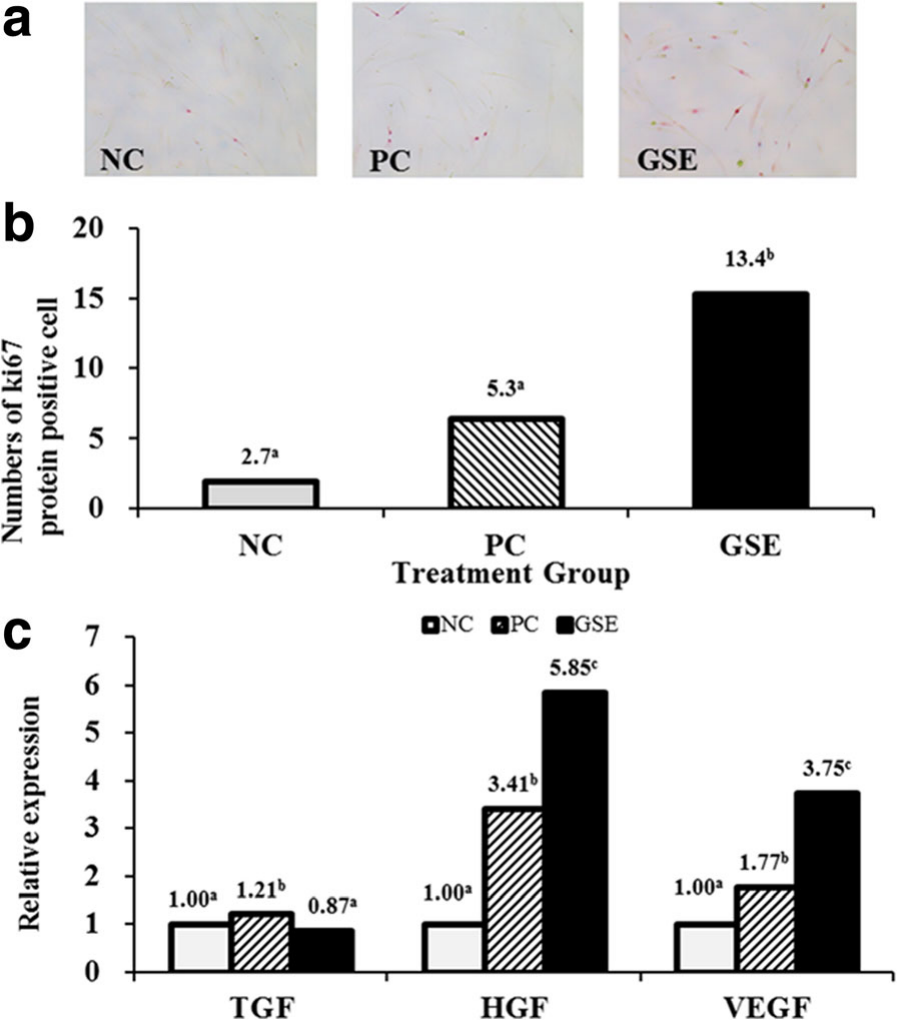
Changes in the levels of Ki67 protein, hepatocyte growth factor (HGF), vascular endothelial growth factor (VEGF), and transforming growth factor beta 1 (TGF-β1) in human dermal papilla cells (hDPCs) induced by GSE. **a** Immunohistochemistry of Ki67. **b** Numbers of Ki67-positive cells. **c** Relative expression levels of HGF, VEGF, and TGF-β1 in GSE-treated hDPCs. The fold changes were normalized to the expression of glyceraldehyde 3-phosphate dehydrogenase; the values are expressed as the mean ± SEM. Values sharing the same superscript letters differ significantly at *p* < 0.05 by Duncan’s multiple range test. NC: negative control of hDPCs treated with Dulbecco’s modified Eagle’s medium; PC: positive control of hDPCs treated with 10 μM minoxidil; GSE: hDPCs treated with 19.5 ppm GSE

Immunohistochemistry localized the expression of Ki-67 protein on hDPCs treated with GSE (Fig. 3a). Positive signal of Ki-67 was observed only in nucleus of hDPCs. Based on the histomorphologic analysis, Ki-67 protein expression was significantly elevated in the GSE-treated group (average 13.4 cells) compared with the NC and PC groups (2.7 and 5.3 cells, respectively) (Fig. 3b).

### mRNA Expression of cytokines in hDPCs

The relative expression levels of VEGF, HGF, and TGF-β1 in GSE-treated hDPCs were measured by real-time RT-PCR (Fig. 3c). Compared with NC group, GSE significantly upregulated mRNA expression of HGF and VEGF in hDPCs by 5.85-fold and 3.75-fold, respectively (*p* < 0.05). Although TGF-β1 mRNA expression of GSE was not significantly different with NC group, PC group significantly increased mRNA expression of TGF-β1 higher than GSE and NC group (*p* < 0.05).

### Histopathology and cytokine expression of depilated mice

**Fig. 4:**
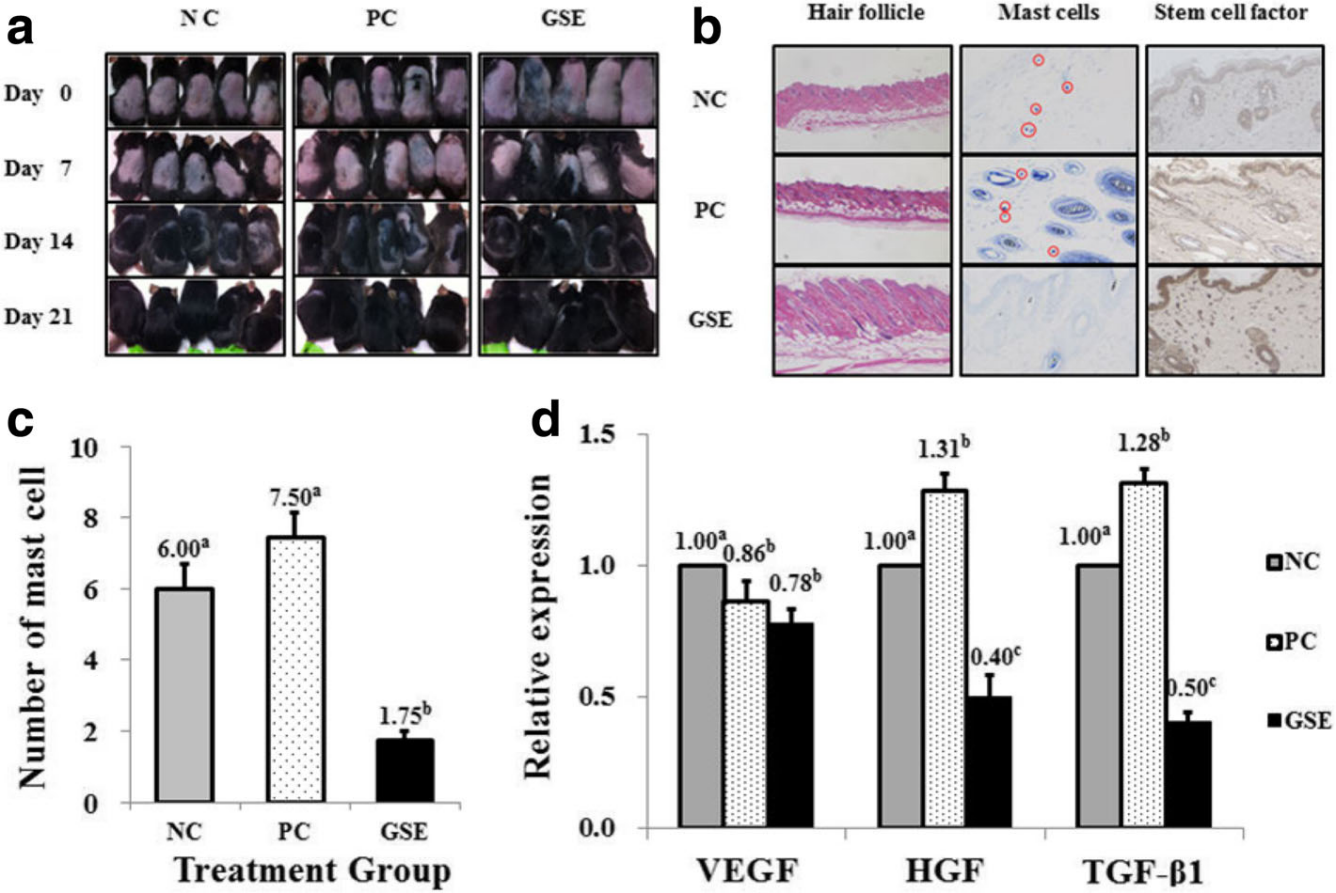
Hair growth-stimulating effect of GSE in an in vivo model. **a** Observation of C57BL/6 mice hair growth for 3 weeks. **b** Histological analysis of hair follicles, mast cells, and stem cell factor expression on the back skin of C57BL/6 mice. **c** The numbers of mast cells were counted by toluidine blue staining. **d** Expression of vascular endothelial growth factor (VEGF), hepatocyte growth factor (HGF), and transforming growth factor beta 1 (TGF-β1) in the back skin of C57BL/6 mice. The fold changes were normalized to the expression of glyceraldehyde 3-phosphate dehydrogenase; the values are expressed as the mean ± SEM. Values sharing the same superscript letters differ significantly at *p* < 0.05 by Duncan’s multiple range test. NC: negative control of C57BL/6 mice skin treated with dimethyl sulfoxide; PC: positive control of C57BL/6 mice skin treated with 5% minoxidil; GSE: C57BL/6 mice skin treated with 1,000 ppm GSE

The morphology of the hair follicles, number of mast cells, and expression of SCF were evaluated by veterinary pathologists (Fig. 4b). From the histological analysis, it was observed that the depth of the hair follicle was deeper, and the length of the hair follicular shaft was longer in the GSE group than those in the NC and PC groups (Fig. 4b). The number of mast cells was significantly lower in the GSE group (1.75 mast cells) than in the NC (6.0 mast cells) and PC (7.5 mast cells) groups (Fig. 4b, c). Mast cells were located in dermal connective tissue. SCF was most strongly expressed at the cytoplasm of skin epidermis and dermal papillary cells of hair follicles in GSE-treated group compared to the NC and PC groups (Fig. 4b). The induction of SCF in the in vivo model suggested that hDPCs underwent the regeneration process of hair follicles.

The mRNA expression of cytokines in mouse skins was measured by qPCR. The expression of HGF, VEGF, and TGF-β1 was normalized with GAPDH housekeeping gene and presented as fold change. The real-time RT-PCR results showed that 1,000 ppm GSE treatment significantly downregulated VEGF, HGF, and TGF-β1 mRNA expression in the mouse skin (Fig. 4d).

## Discussion

Several plants have been widely used for treating or preventing diseases various countries. For instance, GSE has been used in folk remedies to treat diarrhea, bacterial infection, and anti-gastric ulcer agent [2]. Recently, scientific studies have shown that GSE holds pharmacological activity including anti-inflammatory, anti-bacterial function, anti-diarrhea effect and anti-gastric ulcer action as well as high ability of DPPH radical scavenging [11, 12, 14, 15]. However, the effects of extracts from this plant on hair growth have not been studied so far. Therefore, we investigated whether the topical treatment of GSE could promote hair growth, using in vitro and in vivo models.

This study confirmed the corilagin and gallic acid as active components of GSE in HPLC chromatogram as both were identified from *G. sibiricum* [13]. Other study also reported the maximum content of gallic acid in *G. sibiricum* collected in Liaoning Province, China [22]. The antioxidant activity of corilagin and gallic acid was shown by scavenging various radicals effectively [23, 24]. Anti-inflammatory activity and anti-cancer effect of corilagin and gallic acid were also addressed in several studies [25–28]. Furthermore, gallic acid could prevent allergic reaction by blocking histamine release and pro-inflammatory cytokine expression in mast cell [26]. Therefore, it seemed that the action of two major components of GSE contributed the promoting of hair growth by reducing inflammation in vitro and in vivo model.

The total polyphenol and flavonoid contents of GSE in this study were 305.9 mg TAE/g extract and 104 mg NE/g extract, respectively. Their values were significantly higher than those of *Lespedeza cuneata* G. Don (228.9 mg TAE/g extract, 90.2 mg NE/g extract) which was selected by screening the highest antioxidant extract from many plant extracts [29]. Moreover, the electron-donating ability of GSE (76.6%) was not only higher than the synthetic antioxidants butylated hydroxyanisole (69.2%) and butylated hydroxytoluene (45.6%) but also comparable to α-tocopherol (75.1%) and *Lespedeza cuneata* G. Don (75.7%) [29, 30]. Naito et al. [31] has reported that lipid peroxides can induce apoptosis of hair follicle cells and lead to the early onset of catagen phase in murine since it can produce reactive oxygen species (ROS) causing apoptosis of hair matrix and epithelial cells by stimulating the expression of *p*53. Plant extracts with high DPPH radical scavenging activity may prevent hair loss presumably by protecting hair follicle cell from hydroperoxides.

Cell proliferation and migration are the essential factors for hair growth [32–34]. 19.5 ppm of GSE showed cell proliferation and migration of hDPCs significantly higher than those of NC and PC (Fig. 2). Since GSE induced the proliferation and migration of cultured hDPCs possibly, GSE could enhance hair growth positively. However, GSE more than 156.3 ppm caused toxic effect on hDPCs. The induction of Ki-67 in vitro model suggested that GSE treatment activates cell proliferation of hDPCs by maintaining and stimulating the active phases of cell growth, since the Ki67 marker is expressed only in the active phases of cell growth, such as the G1, S, and G2 phases and mitosis [35].

Some cytokines are essential to development, growth and cycling of hair follicles. The results revealed that HGF and VEGF mRNA expression level of hDPCs following treatment with GSE was significantly upregulated compared with NC or PC (*p* < 0.05). Although GSE treatment did not reduce TGF-β1 mRNA expression significantly compared with the NC group, the PC group showed significantly higher mRNA expression of TGF-β1 than the GSE group (1.21 vs. 0.87). Since HGF and VEGF are known growth modulators of the follicular papilla in the anagen stage [34], it stands to reason that the induction of these growth factors by GSE treatment would promote the anagen stage or active hair growth. The downregulation of TGF-β1 may contribute partially to the prevention of hair loss, because this is involved in the catagen stage of hair development or the apoptosis of hair follicle cells [33]. Considering these findings in the in vitro model, GSE treatment may promote the anagen stage of hair growth by modulating growth regulators.

We had also conducted in vivo study to determine hair growth effect of GSE in induced telogenic C57BL/6 mice. From the histological analysis, the depth and development of dermal papillar and the long hair shaft of hair follicles in the GSE group indicated active hair growth rather than in the NC and PC groups. As mast cells play a key role in stress reaction and inflammation, the degranulation of inflammatory mediators by mast cells triggers the catagen stage of hair follicles, intrafollicular apoptosis, and hair loss [2, 36]. In this study, mast cells are observed in dermal connective tissues rather than near hair follicles. Considering a few number of mast cells in GSE group, GSE treatment contribute anti-inflammatory action on dermal tissues rather than on hair follicular epithelium.

Interestingly, this study confirmed that GSE induced SCF in vivo model by immunohistochemistry. SCF was strongly expressed in mouse epidermal keratinocytes and epithelium of hair follicular bulbs. Since the SCF plays a role in preserving stem cells and participating in the growth of hair follicles, it is detectable in the anagen stage [37]. The induction of endothelin-1 and SCF was correlated with the regeneration of hair follicles [38]. In concurrence with the previous studies, the strong induction of SCF in the GSE-treated mice skin suggested that hDPCs underwent the regeneration process of hair follicles.

Interestingly, the treatment of 1,000 ppm GSE significantly downregulated mRNA expression of VEGF, HGF, and TGF-β1 in the mouse skin (Fig. 4d). While GSE upregulated expression of HGF and VEGF on hDPCs, the expression of HGF and VEGF was downregulated in mouse skin tissues. Since induction of VEGF and HGF were required in active anagen phase, they are expressed in actively growing hair follicles. As 24 h of GSE treatment induced anagen phase of hDPCs, expression of VEGF and HGF was upregulated in GSE-treated hDPCs. However, hair follicles in mouse skin tissues after 3 weeks of depilation were in catagen phase rather than anagen when it is considered that GSE-treated group finished the hair growth faster than PC or NC. Therefore, catagen phase could explain the reduced expression of VEGF and HGF in GSE-treated mouse hair follicles for 3 weeks.

It was surprising finding that TGF-β1 was downregulated in both models. As the function of TGF-β1 is associated with hair loss, upregulation of TGF-β1 in hair growth inhibits the growth of hair follicular cells by accelerating the transition of catagen and telogen. Thus, downregulation of TGF-β1 could prevent hair loss in GSE-treated skin by maintaining catagen phase of hair follicles and viability of follicular cells. Otomo [39] reported that minoxidil inhibits of TGF-β induced apoptosis of hair matrix cells by opening the Kir 6.0 channel pore coupled with sulfonylurea receptor on the mitochondrial inner membrane. The downregulation of TGF-β1 in both in vitro and in vivo models indicated that GSE may have the same mechanism with minoxidil in hair growth activity. Although corilagin and gallic acid were identified as active components in GSE, there was a limitation that hair growth effect of each chemical was not measured. Therefore, their hair growth effect and mechanism should be investigated in further study.

## Conclusions

This study confirmed that GSE treatment significantly promoted hair growth, both in vitro and in vivo, by the proliferation and enhanced migration of hDPCs. GSE treatment also modulated the expression of HGF, VEGF, and TGF-β1, which are involved in the growth and inhibition of hair follicular cells. The reduced number of mast cells and the induction of SCF also contributed to hair regeneration events. Therefore, we concluded that GSE could enhance hair growth and prevent hair loss.

## Abbreviations

ACN: Acetonitrile
CCK-8: Cell Counting Kit-8
DEX: Dexamethasone
DMEM: Dulbecco’s modified Eagle’s medium
DMSO: Dimethylsulfoxide
EDA: Electron donating ability
GAPDH: Glyceraldehyde 3-phosphate dehydrogenase
GSE: Geranium sibiricum L. extract
hDPCs: Human dermal papilla cells
HGF: Hepatocyte growth factor
HPLC: High pressure liquid chromatography
MeOH: Methanol
MXD: Minoxidil
NC: Negative control, NE, naringin equivalent
PC: Positive control
qPCR: Quantitative real-time polymerase chain reaction
SCF: Stem cell factor
TAE: Tannic acid equivalent
TGF-β1: Transforming growth factor-beta 1
VEGF: Vascular endothelial growth factor

## References

[1] So HR, We SY, Im EJ (2011). Comparison of hair loss factors by sex in Seoul and Chungcheon area -Comparison of hair loss factors by sex-. J Korean Soc Cosmetol.

[2] Arca E, Açikgöz G, Taştan HB, Köse O, Kurumlu Z (2004). An open, randomized, comparative study of oral finasteride and 5% topical minoxidil in male androgenetic alopecia. Dermatology.

[3] McCellan KJ, Markham A (1999). Finasteride; A review of its use in male pattern hair loss. Drugs.

[4] Trueb RM, Itin P (2001). Photographic documentation of the effectiveness of 1 mg oral finasteride in treatment of androgenic alopecia in the man in routine general practice in Switzerland. Praxis.

[5] Sharquie KE, Al-Obaidi HK (2002). Onion juice (*Allium cepa* L.), a new topical treatment for alopecia areta. J Dermatol.

[6] Yoon JI, Al-Reza SM, Kang SC (2010). Hair growth promoting effect of *Zizyphus jujuba* essential oil. Food Chem Toxicol.

[7] Leonard BE (2005). The HPA, and immune axes in stress: the involvement of the serotonergic system. Eur Psychiatry.

[8] Paus R, Langan EA, Vidali S, Ramot Y, Andersen B (2014). Neuroendocrinology of the hair follicle: principles and clinical perspectives. Trends Mol Med.

[9] Zhang L, Ravipati AS, Koyyalamudi SR, Jeong SC, Reddy N, Smith PT, Bartlett J, Shanmugam K, Munch G, Wu MJ (2011). Antioxidant and anti-inflammatory activities of selected medicinal plants containing phenolic and flavonoid compounds. J Agric Food Chem.

[10] Wu N, Zu Y, Fu Y, Kong Y, Zhao J, Li X (2010). Antioxidant activities and xanthine oxidase inhibitory effect of extract and main polyphenolic compounds obtained from *Geranium sibiricum* L. J Agric Food Chem.

[11] Lee SE, Seong NS, Bang JK, Park CG, Park JS, Sung J (2003). Antioxidative activities of Korean medicinal plants. Korean J Med Crop Sci.

[12] Fan HY, Guo JP, Yu HL (2006). Effects of *Geranium sibirium* extract on gastric ulcer induced by indomethacin and reserpine in mice. J Med Sci Yanbian Univ.

[13] Guo JS, Wang SX, Li X, Zhu TR (1987). Studies on the antibacterial constituents of *Geranium sibiricum* L. Acta Pharm Sin.

[14] Shim JU, Lim KT (2009). Antioxidative activity of glycoprotein isolated from *Geranium sibiricum* Linne. Nat Prod Res..

[15] Wang L, Liu J, Lu Y, Yang L, Yang L, Fu Z, Tan H (2004). Effects of Geranium sibiricum L. on MDA content and SOD activity in intestinal mitochondrion of the diarrhea mice. Heilongjiang Med Pharm.

[16] Shim JU, Oh PS, Lim KT (2009). Anti-inflammatory activity of ethanol extract from *Geranium sibiricum* Linne. J Ethnopharmacol.

[17] Inui S, Itami S (2013). Androgen actions on the human hair follicle: perspectives. Exp Dermatol.

[18] Blois MS (1958). Antioxidant determinations by the use of a stable free radical. Nature.

[19] Boyera N, Galey I, Bernard BA (1997). Biphasic effects of minoxidil on the proliferation and differentiation of normal human keratinocytes. Skin Pharmacol Physiol.

[20] Evans CP, Elfman F, Cunha G, Shuman MA (1997). Decreased prostate cancer cell migration by inhibition of the insulin-like growth factor II/Mannose-6-Phosphate receptor. Urol Oncol.

[21] Choi SJ, Cho AR, Jo SJ, Hwang ST, Kim KH, Kwon OS (2013). Effects of glucocorticoid on human dermal papilla cells in vitro. J Steroid Biochem Mol Biol.

[22] He J, Kang T, Yin H, Zheng Y, Zhang S (2007). The content determination of gallic acid of *Geranium sibiricum* collected in three Provinces in Northeast. Chin Arch Tradit Chin Med.

[23] Kinoshita S, Inoue Y, Nakama S, Ichiba T, Aniya Y (2007). Antioxidant and hepatoprotective actions of medicinal herb, *Terminalia catappa* L. from Okinawa Island and its tannin corilagin. Phytomedicine.

[24] Yen GC, Duh PD, Tasi HL (2002). Antioxidant and pro-oxidant properties of ascorbic acid and gallic acid. Food Chem.

[25] Faried A, Kurnia D, Faried LS, Usman N, Miyazaki T, Kato H (2007). Anticancer effects of gallic acid isolated from Indonesian herbal medicine, *Phaleria macrocarpa* (Scheff.) Boerl, on human cancer cell lines. Int J Oncol.

[26] Kim SH, Jun CS, Suk K, Choi BJ, Lim H, Park S (2006). Gallic acid inhibits histamine release and pro-inflammatory cytokine production in mast cells. Toxicol Sci.

[27] Jia L, Jin H, Zhou J, Chen L, Lu Y, Ming Y (2013). A potential anti-tumor herbal medicine, Corilagin, inhibits ovarian cancer cell growth through blocking the TGF-β signaling pathways. BMC Complement Altern Med.

[28] Jin F, Cheng D, Tao JY, Zhang SL, Pang R, Guo YJ (2013). Anti-inflammatory and anti-oxidative effects of corilagin in a rat model of acute cholestasis. BMC Gastroenterol.

[29] Kim EJ, Choi JY, Yu MR, Kim MY, Lee SH, Lee BH (2012). Total polyphenols, total flavonoid contents, and antioxidant activity of Korean natural and medicinal plants. Korean J Food Sci Technol.

[30] von Gadow A, Joubert E, Hansmann CF (1997). Comparison of the antioxidant activity of aspalathin with that of other plant phenols of rooibos tea (*Aspalathus linearis*), α-tocopherol, BHT, and BHA. J Agric Food Chem.

[31] Nait A, Midorikawa T, Yoshino T, Ohdera M (2008). Lipid peroxides induce early onset of catagen phase in murine hair cycles. Int J Mol Med.

[32] Alonso L, Fuchs E (2006). The hair cycle. J Cell Sci.

[33] Botchkarev VA, Kishimoto J (2003). Molecular control of epithelial-mesenchymal interactions during hair follicle cycling. J Investig Dermatol Symp Proc.

[34] Philpott MP, Kealey T (1994). Effects of EGF on the morphology and patterns of DNA synthesis in isolated human hair follicles. J Investig Dermatol.

[35] Rahmanzadeh R, Huttmann G, Gerdes J, Scholzen T (2007). Chromophore-assisted light inactivation of pKi-67 leads to inhibition of ribosomal RNA synthesis. Cell Prolif.

[36] Maurer M, Paus R, Czarnetzki BM (1995). Mast cells as modulators of hair follicle cycling. Exp Dermatol.

[37] Huelsken J, Vogel R, Erdmann B, Cotsarelis G, Birchmeier W (2001). β-catenin controls hair follicle morphogenesis and stem differentiation in the skin. Cell.

[38] Lü ZF, Cai SQ, Wu JJ, Zheng M (2006). Biological characterization of cultured dermal papilla cells and hair follicle regeneration *in vitro* and *in vivo*. Chin Med J.

[39] Otomo S (2002). Hair growth effect of minoxidil. Nihon Yakurigaku Zasshi.

